# Protocol for the real-world evidence of acupuncture for chronic neck pain in the elderly: an observational multi-centre cohort study

**DOI:** 10.3389/fmed.2026.1737161

**Published:** 2026-06-30

**Authors:** Anzhe Tian, Mailan Liu, Mengxia Xiao, Chao Liu, Cheng Guo, Xinran Xiao, Saiyang Liu, Xiang Li

**Affiliations:** 1 College of Acupuncture, Tuina and Rehabilitation, Hunan University of Chinese Medicine, Changsha, China; 2 The First Hospital of Hunan University of Chinese Medicine, Changsha, China

**Keywords:** acupuncture, aging, chronic pain, cohort study, neck pain, protocol, real-world

## Abstract

**Introduction:**

Chronic neck pain (CNP) refers to persistent pain lasting more than 3 months originating from the muscles, bones, joints, or tendons in the cervical region. With global population growth and the accelerating trend of aging, the number of elderly patients suffering from neck pain is continuously increasing, and the associated health burden and socioeconomic costs cannot be overlooked. Previous studies have shown that acupuncture therapy in Traditional Chinese Medicine (TCM) is effective in alleviating pain among elderly patients with CNP. However, few studies have investigated the overall effectiveness of various acupuncture therapies in real-world clinical settings for managing CNP in the elderly.

**Methods and analysis:**

A total of 212 eligible older adults with CNP will be enrolled and classified into an acupuncture group or a non-acupuncture group according to routine clinical practice, patient preference, and physician judgment. All participants will receive treatment three times per week for 4 weeks, followed by an 8-week follow-up period. The primary outcome will be the proportion of patients with at least 50% reduction from baseline in the worst pain intensity measured by Visual Analogue Scale (VAS) (0–100, higher scores signify worse pain) at baseline and after treatment. Secondary outcomes include changes in the Northwick Park Neck Pain Questionnaire (NPQ) score, changes in the Tanaka Yasuhisa 20-point assessment scale, and alterations in the cervical pressure pain threshold (PPT). VAS, NPQ and Tanaka Yasuhisa 20-point score will be assessed at weeks 0, 4, 8, and 12. PPT will be measured at weeks 0 and 4. Adverse events will be recorded in detail during the trial.

**Ethics and dissemination:**

The study has been approved by our ethics review boards (Protocol Approval HN-LL-KY-2025-009-01, 2025-KY-019-01, No. 2025042704). The study findings will be disseminated through presentation at a high-impact medical journal, with online access.

**Conclusion:**

The findings of this study will provide potential evidence for manual acupuncture in the treatment of CNP.

**Clinical trial registration:**

https://itmctr.ccebtcm.org.cn, ITMCTR2025002064.

## 1 Introduction

Chronic neck pain (CNP) is defined as persistent pain lasting more than 3 months originating from the muscles, bones, joints, or tendons in the cervical region (1). The annual prevalence of neck pain exceeds 30%, and nearly 50% of patients with acute neck pain will develop persistent symptoms (2). Globally, the prevalence of CNP is higher in women than in men, peaking among those aged 45 to 74. Furthermore, amid global population growth and an accelerating aging trend, the number of elderly patients with neck pain is continually increasing, and the associated health burden and socioeconomic costs cannot be overlooked (3). CNP not only affects patients’ physical function, quality of life, and work productivity but can also lead to negative emotions such as anxiety and depression, severely impairing overall wellbeing (4). Additionally, it contributes to increased direct medical costs and indirect societal expenses. In the United States, neck pain ranks first in annual healthcare spending, amounting to as much as $134.5 billion (5).

The exact pathogenesis of CNP remains unclear, and the underlying mechanisms responsible for its persistence, recurrence, and progression are still not fully understood. Research evidence indicates that (6) poor posture, sleep disturbances, obesity, prolonged computer use, anxiety, depression, and other negative emotional states are significant risk factors for CNP. Among these, poor posture and psychological issues are closely associated with CNP (7, 8). Maintaining poor posture over extended periods can lead to abnormal movement patterns in the cervical spine, resulting in alterations in the motor and sensory systems of the neck (9). These changes include modified activation patterns of neck muscles, impaired proprioception, reduced muscle endurance, decreased range of motion, and diminished coordination.

Clinically, a variety of approaches are available for managing CNP. Patients with severe pain may use nonsteroidal anti-inflammatory drugs (NSAIDs) (10). Those with predominant muscle pain in the neck and shoulder region may benefit from physical agent therapies such as sound, light, electrical, magnetic, and heat-based modalities—which are also considered effective methods for treating chronic non-specific neck pain (11–14). For patients experiencing limited mobility in the neck and shoulder areas, exercise therapy can be applied to improve the range of motion (15). Traction therapy may be used in cases where cervical spine dysfunction is evident. For patients with long-lasting and severe pain, injection therapy and surgical intervention may be considered (12).

In Traditional Chinese Medicine (TCM), CNP falls under the category of “neck Bi.” Its pathogenesis is primarily attributed to Qi stagnation and blood stasis in the meridians, which leads to pain due to obstruction. Over time, this results in malnourishment of the tendons and vessels, causing pain due to lack of nourishment (16). TCM has accumulated extensive experience in preventing and treating CNP, with external therapies playing a crucial role in its management. Through pattern differentiation and treatment approaches such as tuina (therapeutic massage) and acupuncture, TCM has provided viable pathways for alleviating pain and improving quality of life (17).

Currently, acupuncture is recognized as one of the safe and effective mainstream treatments for CNP (18). According to TCM theory, acupuncture acts by stimulating specific points on the body to regulate the flow of qi and blood in the meridians and harmonize the function of internal organs, thereby preventing and treating diseases. From a modern medical perspective, acupuncture alleviates pain by releasing adhesions, improving local blood circulation, and reducing muscle spasms (19–21). Acupuncture stimulates specific acupoints, prompting the nervous system to release neurotransmitters such as endorphins and *β*-endorphin, which exert analgesic effects (22). Simultaneously, acupuncture enhances local blood flow, relieving muscle tension and stiffness in the neck, improving cervical mobility, and reducing discomfort (23).

To the best of our knowledge, most clinical studies on acupuncture for CNP have been conducted in the form of randomized controlled trials (RCTs), and no related real-world studies have been conducted to date. Therefore, this study aims to investigate the overall clinical effectiveness of acupuncture therapy in elderly patients with CNP through a prospective, multi-center, real-world study.

## 2 Methods and design

### 2.1 Study design

**Figure 1:**
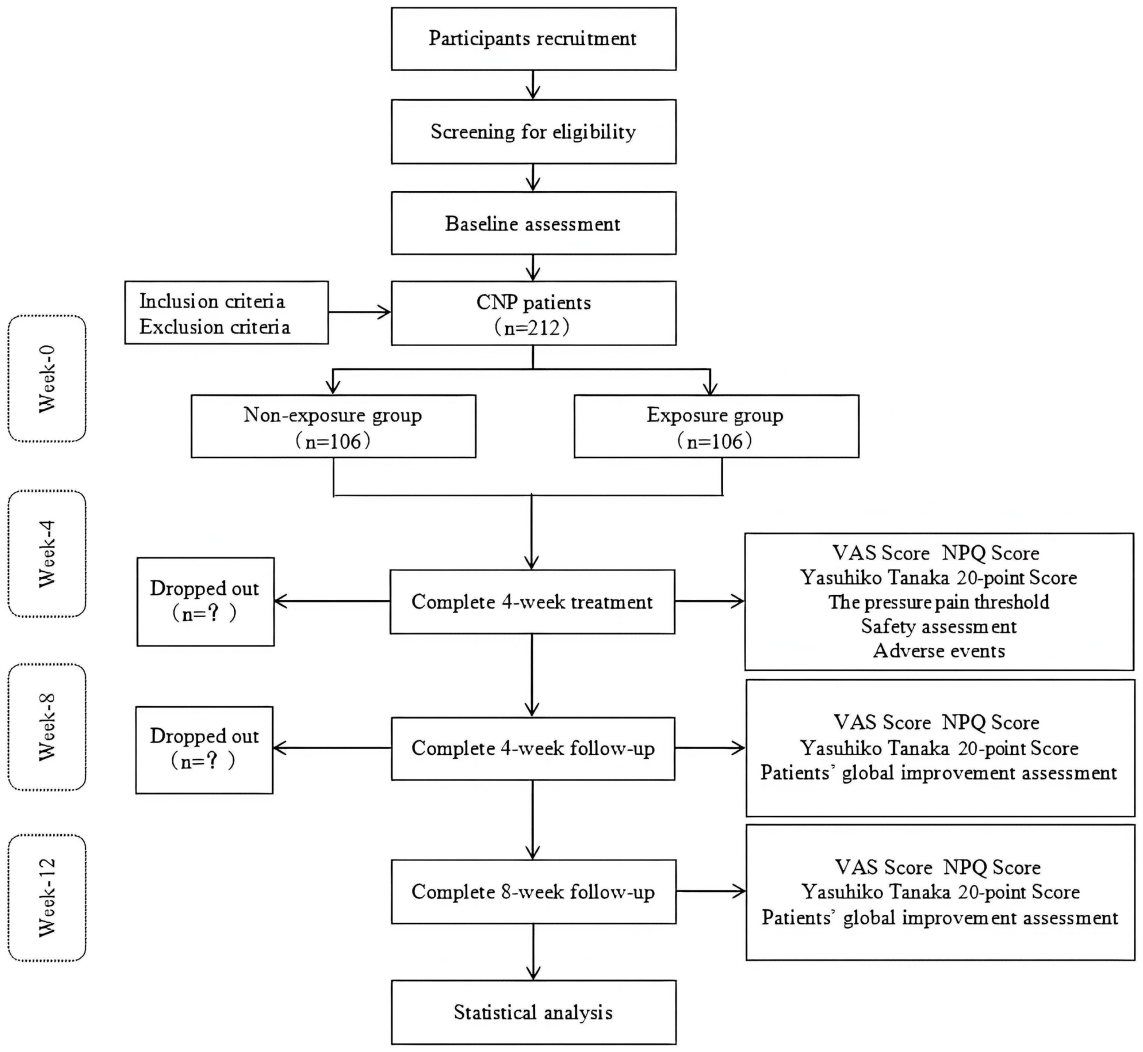
Flowchart of the study.

We will conduct a multi-center, prospective, observational real-world study. Elderly patients with CNP who meet the diagnostic and inclusion criteria will be enrolled from the outpatient departments of three participating hospitals. All patients must understand and sign an informed consent form before participation. The flowchart is shown in Figure 1 and the time points of assessment are shown in Table 1.

**Table 1:** Schedule of data collection.

	**Study period**			
	**Inclusion**	**Treatment**	**Follow-up**	
TIME POINT(W,week)	WO	W4±2d	W8±2d	W12±2d
Assessments	Baseline	Second	Third	Fourth
Eligibility criteria	V			
Demography characteristics	V			
Eligibility screen	V			
Informed consent	V			
Interventions	W1-W4			
Treatment		V		
VAS Scale	V	V	V	V
NPQ Scale	V	V	V	V
Yasuhiko Tanaka 20-point Scale	V	V	V	V
The pressure pain threshold	V	V		
Patients' global improvement assessment		V	V	V
Patients' expectations for acupuncture	V			
Safety assessment		V	V	V
Adverse events		V	V	V

### 2.2 Study setting and recruitment

This trial will be performed at The First Hospital of Hunan University of Chinese Medicine, The Second Hospital of Hunan University of Chinese Medicine and Changsha Hospital of Traditional Chinese Medicine (Changsha Eighth Hospital) between December 2025 and December 2026. A total of 212 participants will be recruited through posters, hospital websites and networks. The study duration for each participant will be 13 weeks: a 1-week baseline, 4 weeks of treatment, and an 8-week follow-up.

### 2.3 Patient and public involvement

No patient involved.

### 2.4 Participants

#### 2.4.1 Inclusion criteria

1. Having CNP for more than three months.
2. A minimum score of 40 in worst pain intensity according to the 100-point visual analogue scale (VAS).
3. Age 60–75 years old.
4. Signed the informed consent prior to the inclusion.

#### 2.4.2 Exclusion criteria

1. Have undergone cervical spine surgery within the past year.
2. Neurological or systemic diseases including rheumatoid arthritis, diabetes, cardiovascular disorder, severe hepatic/renal insufficiency or coagulation disorder.
3. Needle-phobic patients or had received acupuncture in the past 4 weeks.
4. Cognitive impairments which restrict them from understanding commands, evidenced by their medical diagnosis or any kind of psychiatric disorder.
5. Participants unwilling or unable to comply with study schedules or follow-up assessments.

#### 2.4.3 Allocation

Elderly individuals with CNP will be recruited through posted flyers, online advertisements, and physician referrals. Hospital research assistants will conduct initial screenings to document participants’ medical conditions, history, and study-related baseline data. Potential candidates will undergo a 7-day baseline assessment, and those who meet the trial criteria will be enrolled. Acupuncturists will administer treatments to the participants and, in collaboration with other researchers, guide them in completing trial-related documentation. Investigators at each sub-center will accurately record patient data in case report forms. After enrollment, patients will receive standard care and will be divided into a non-acupuncture group and an acupuncture group based on either patient preference or physician-determined treatment plans.

Data will be entered into the clinical research data management platform at four time points. The first visit: before treatment initiation after hospital admission (Day 0); The second visit: after completion of treatment courses (Week 4); The third visit is conducted at 4 weeks post-treatment, corresponding to a 4-week follow-up (Week 8), and the fourth visit at 8 weeks post-treatment, corresponding to an 8-week follow-up (Week 12). Data will then compiled and analyzed.

#### 2.4.4 Non-acupuncture group

The non-acupuncture group will receive non-acupuncture treatments, including oral pharmacological therapy, rehabilitation therapy, and tuina (therapeutic massage). Older adults with CNP who meet the diagnostic and eligibility criteria will, after consultation with a specialist physician, continue their existing non-acupuncture treatment regimens without modification.

1. Western medicine treatments may include non-steroidal anti-inflammatory drugs (e.g., meloxicam), neurotrophic agents (e.g., mecobalamin tablets), and glucocorticoids.
2. Chinese herbal medicine will be prescribed based on traditional Chinese medicine syndrome differentiation, with modified formulations such as *Guizhi Fuzi Tang*, *Huoxue Zhitong Tang*, *Qianghuo Shengshi Tang*, *Duhuo Jisheng Tang*, and *Guipi Tang*.
3. Rehabilitation therapy may include physical agent modalities, traction therapy, manual therapy, exercise therapy, and the application of orthotic devices.
4. Tuina therapy will primarily involve soft-tissue relaxation techniques and orthopedic manipulation, with cervical mobilization applied when appropriate.

Detailed treatment information will be recorded, including the total number of treatment sessions, treatment frequency, medication names, dates of administration, routes of administration, and treatment duration.

#### 2.4.5 Acupuncture group

The acupuncture group will receive acupuncture-based interventions, including manual acupuncture, fire needling, warm needling, auricular acupuncture, abdominal acupuncture, and acupotomy.

Acupuncture will be administered by licensed acupuncturists who have undergone formal theoretical and practical training. The selection of acupoints follows patterns identified in clinical literature, focusing on the 10 most frequently used points in acupuncture prescriptions for cervical spondylosis. These include: *Jingjiaji* (EX-B2), bilateral *Tianzhu* (BL10), bilateral *Houxi* (SI3), bilateral *Shenmai* (BL62), and *Ashi* points(the most tender point of the neck). The location of the acupoints will be based on Nomenclature and location of acupuncture points (24) drafted in 2006 by the National Standard of the People’s Republic of China (GB/T12 346-2006).

During acupuncture treatment, the patient assumes a prone position. The treatment area and the practitioner’s hands are routinely sterilized with 75% alcohol. Disposable sterile Huatuo brand acupuncture needles (0.30 mm × 40 mm) are used. After the local skin was routinely sterilized, the participants’ Ashi points will be vertically inserted by the needles to a depth of 10–15 mm. Manipulation via lifting, thrusting, twirling, and rotating techniques will be performed to achieve de qi (25) (the requisite sensation encompassing sourness, numbness, swelling, and heaviness). Upon achieving the deqi sensation, a balanced reinforcing-reducing manipulation technique is applied. For EX-B2, emphasis is placed on obtaining a needle sensation that propagates to the affected shoulder, upper back, and forearm. The needles are retained for 30 min. Acupuncture therapy is administered once daily, three times per week. Six sessions constitute 1 treatment course, the total intervention period consists of 2 courses.

Document comprehensive details of acupuncture treatment, including the specific acupuncture techniques employed, treatment protocols, number of needles used, modes of stimulation, course of therapy, treatment frequency, and overall duration of the intervention.

### 2.5 Outcome measures

#### 2.5.1 Primary outcome

The primary outcome will be the reduction in VAS Score from Baseline: Upon admission, patients will be asked to complete a Visual Analog Scale (VAS) to assess their pain level, which will serve as the baseline measurement. The VAS consists of a 10-cm horizontal line, anchored at both ends by verbal descriptors ranging from “no pain” to “the most severe pain imaginable.” Scores are interpreted as follows: 0 indicates no pain; 10–30 mild pain; 40–60 moderate pain; 70–90 severe pain; and 100 represents the worst pain possible. After completing the treatment, subjects will be asked to rate their average pain level over the past week using the same VAS. The reduction in the VAS score from baseline will be calculated and used as the primary outcome measure.

#### 2.5.2 Secondary outcomes

The secondary outcomes include the following items:

1. Changes in the Neck Pain Questionnaire (NPQ) scores after 4-week treatment and weeks 8, weeks 12. The NPQ includes 9 items, each scored from 0 to 4, with higher total scores indicating worse neck condition.
2. Changes in the Yasuhiko Tanaka 20-point Scale after 4-week treatment and weeks 8, weeks 12. This scale evaluates the severity of cervical spondylosis across four domains: symptoms (neck/shoulder pain and discomfort, upper limb pain and/or numbness, finger pain and/or numbness), work and daily living capacity, physical signs (Spurling’s test, sensation, muscle strength, tendon reflexes), and hand function. The total score is 20, with lower scores indicating more severe cervical spondylosis.
3. The pressure pain threshold (PPT) reflects the sensitivity of muscle tissue to nociceptive stimuli. In this study, the PPT will be recorded in Newton/cm^2^ using the digital algometer (Force Ten™-Model FDX, Wagner, Greenwich, USA) with a surface area of round tip of 1 cm^2^. The PPT will be assessed at two muscle sites, the upper trapezius and the sternocleidomastoid. The midpoint of the muscle belly of each muscle will serve as the measurement location. Participants will be assessed in a seated position. For the upper trapezius, the point corresponds to *Jingjiaji* (EX-B2), located at the midpoint between the C7 spinous process and the acromion. For the sternocleidomastoid, the point is located at the midpoint between the mastoid process and the sternoclavicular joint, approximately at the level of the fourth cervical vertebra. During the measurement, participants will be instructed to indicate when they first perceive a slight pain sensation. The examiner will position the probe perpendicular to the test site and gradually apply pressure while monitoring the digital display. Once the participant signals pain, pressure will be released immediately and the value recorded. Each site will be measured three times, and the average value will be used for analysis. PPT values obtained before and after treatment will be documented to assess changes in muscle pain sensitivity.
4. Use of analgesic medication: The types, frequency, and dosage of medications used will be recorded.
5. Surgery: Whether the patient underwent surgery due to pain will be documented.
6. Hospitalization: Whether the patient was hospitalized due to pain will be documented.
7. Re-consultation status: Whether the patient sought additional medical care for the same pain condition (specific criterion: a follow-up visit for the same reason after being determined to be in a state of recovery) will be recorded.
8. Patients’ global improvement assessment: Patients’ global improvement will be assessed by a 7-point self-reporting scale ranging from 1 to 7, where one indicates “complete recovery,” two indicates “obvious improvement,” three indicates “a little improvement”, four indicates “no change”, five indicates “a little worse”, six indicates “obviously worse” and seven indicates “vastly worse.” The proportions of participants in each category of global improvement assessment will be measured after the 4-week treatment and weeks 8, weeks 12.

### 2.6 Data collection

#### 2.6.1 Basic information of the research center and physicians

Research center: name, accreditation level, location, and type of institution.

Physicians: name, age, gender, place of residence, contact information, professional title, and years of experience.

#### 2.6.2 Patient baseline information

Demographic characteristics: age, gender, place of residence, insurance status, education level, smoking status, alcohol consumption, employment status, occupational category, self-rated health status, and physical activity level.

Medical history: classification and location of chronic pain conditions, pain intensity, level of functional impairment, history of sick leave due to pain, duration of condition, previous acupuncture treatment, history of past pain treatments, and concomitant medication use (including psychotropic drugs, NSAIDs, opioids, and other analgesics).

#### 2.6.3 Safety assessment

If an adverse event occurs, appropriate and timely medical management will be provided and the event will be recorded in the Case Report Form (CRF). If a severe adverse event occurs, the intervention will be discontinued and the participant may be withdrawn from the study. Acupuncture-related adverse events, such as fainting during acupuncture, will be managed by immediately stopping needling, removing all needles, placing the patient in a supine position, and maintaining warmth. Minor subcutaneous bleeding or bruising usually resolves spontaneously, while significant swelling, severe pain, or extensive bruising will be managed with cold compression and appropriate symptomatic treatment. For moxibustion-related adverse events, mild skin redness or local warmth generally requires observation only. If blistering or burns occur, moxibustion will be stopped immediately and appropriate wound care will be provided. For massage (tuina)-related adverse events, temporary soreness or mild discomfort will be managed with rest or symptomatic treatment. If significant pain or tissue injury occurs, the treatment will be discontinued and appropriate medical care will be provided. For oral medication-related adverse events, such as gastrointestinal discomfort, nausea, or allergic reactions, symptomatic treatment will be administered and the medication may be discontinued if necessary. All adverse events will be documented in the CRF, including the time of occurrence, severity, duration, management measures, and outcomes. The relationship between the adverse event and the intervention will be assessed using a standard five-category classification system, and severity will be graded according to Good Clinical Practice (GCP) guidelines. All adverse events will be followed until resolution or stabilization. Serious adverse events will be reported and managed in accordance with GCP requirements.

#### 2.6.4 Sample size calculation

This study is an exploratory real-world investigation, and no direct preliminary data are available to support an accurate sample size calculation. Therefore, we referred to the findings of a recent randomized controlled trial (26). In the study, the expected difference in the primary outcome between the acupuncture and non-acupuncture groups was 0.605, with a pooled standard deviation of 1.48, corresponding to an effect size (Cohen’s *d*) of 0.41. Sample size estimation is conducted using G*Power version 3.1.9.7. For a two-independent-samples *t*-test with a two-sided significance level of *α* = 0.05, 80% statistical power (1 − *β* = 0.80), and a 1:1 allocation ratio, the required sample size was 95 participants per group. Considering a potential dropout rate of 10%, the adjusted sample size is approximately 106 participants per group, yielding a total planned enrollment of about 212 participants.

### 2.7 Statistical analysis

Statistical analyses will be performed using SPSS 25.0. Continuous variables will first be tested for normality. Normally distributed data will be expressed as mean ± standard ( $\bar{x}$ ± s) deviation and analyzed using independent-samples t tests or analysis of variance (ANOVA), while non-normally distributed data will be presented as median (P25, P75) and analyzed using nonparametric tests. Categorical variables will be expressed as frequencies and percentages and analyzed using the chi-square test or Fisher’s exact test.

The primary analysis will follow the intention-to-treat (ITT) principle. Missing data will be handled using multiple imputation methods where appropriate. To reduce potential confounding in this real-world observational study, multivariable regression models will be used to adjust for baseline covariates. Repeated measurements at different time points will be analyzed using generalized estimating equations (GEE) or mixed-effects models. Multiplicity adjustment will be performed for multiple secondary outcomes when necessary. A two-sided *p* value < 0.05 will be considered statistically significant.

### 2.8 Quality control

Prior to the commencement of the study, all involved personnel will receive standardized training to ensure thorough familiarity with the study protocol, clinical procedures of acupuncture, precautions, and the management of adverse events. All operators across centers must possess a valid acupuncture practitioner certification and have at least 2 years of independent clinical experience.

Each center will strictly adhere to the diagnostic, inclusion, and exclusion criteria during patient screening. The study will uphold the principles of informed consent and voluntary participation. Subjects with poor compliance will be excluded or withdrawn from the study. Efforts will be made to maintain a positive physician-patient relationship, effective communication, a comfortable medical environment, and reasonable costs to ensure high compliance throughout the study.

A uniform and standardized case observation form will be developed to comprehensively and systematically evaluate the clinical efficacy of the subjects.

All research materials, including the original integrated database and the cleaned research database, will be maintained by designated team members to ensure data security.

The results obtained from this study will be submitted for publication in medical journals. However, all subject information will be kept strictly confidential in accordance with legal requirements. Personally identifiable information will not be disclosed unless required by law.

### 2.9 Ethics and dissemination

This study will be conducted in accordance with the Declaration of Helsinki. Ethical approval has been obtained from the Ethics Committees of all participating centers, and written informed consent will be obtained from all participants prior to enrolment. The investigator must provide detailed information to participants in writing regarding the trial’s purpose, procedures, potential benefits, possible risks, and group allocation. Participants will be informed that participation is voluntary and that they may withdraw at any time without affecting their medical rights and benefits.

All personal information will be kept strictly confidential. Access to medical records for verification purposes will be limited to the investigators, monitors, ethics committee, and regulatory authorities within the bounds permitted by law. The findings of this study will be disseminated through publication in a high-impact, peer-reviewed medical journal with open online access. Additionally, the results are planned to be presented at relevant international conferences and scientific meetings following the publication of the trial outcomes.

## 3 Discussion

The findings of this study will help clarify the effectiveness of acupuncture therapy in managing CNP among the elderly within a real-world clinical setting. Although previous RCTs have demonstrated the efficacy of certain acupuncture for CNP, there remains a notable lack of large-scale, multi-center observational studies that reflect routine practice, particularly in elderly populations (27–31). This study aims to bridge this gap by evaluating the real-world effectiveness of acupuncture therapy, thereby offering insights that are directly applicable to everyday clinical settings.

The study focuses on CNP in the elderly population. Research indicates that 62% of individuals aged 75 years and above suffer from long-term chronic pain, a prevalence rate significantly higher than that observed in younger and middle-aged groups (32). Characterized by high incidence and diverse manifestations, CNP substantially impacts the health status and quality of life of older adults (33). Thus, the treatment of CNP in the elderly urgently requires greater attention.

The VAS is one of the most widely used and validated tools for assessing pain intensity. It has been consistently employed in studies evaluating neck pain and related interventions (34). Since pain severity and functional limitation are core concerns in CNP, the primary outcome of this study is defined as the proportion of patients achieving at least a 50% reduction from baseline in worst pain intensity measured by VAS after two treatment courses. This threshold is clinically significant and aligns with recommendations for interpreting meaningful change in pain trials.

As a secondary outcome measure, this study will compare changes in patients’ PPT before and after treatment. CNP, being a subjective pain experience influenced by physiological, psychological, personal history, and sociocultural factors, is often assessed using methods that are highly subjective, complex in application, and challenging to implement clinically (35). These conventional approaches rely heavily on individual perception and are susceptible to recall bias and state-dependent variability, lacking objective and quantifiable standards. The pressure pain threshold, defined as the minimum pressure that elicits pain when applied to muscle tissue, reflects the degree of muscle tension and sensitivity. It also serves as an indicator for assessing muscle fatigue and potential tissue injury. Compared with traditional subjective scales, this measure provides a relatively objective assessment of changes in patients’ pain perception.

This study has several strengths, including its multi-center, prospective real-world design, relatively large sample size, use of standardized and validated outcome tools, intention-to-treat analysis, and structured quality control procedures. Furthermore, the study reflects routine clinical practice, enhancing the generalizability of its results.

However, several limitations should be acknowledged. The non-randomized design may introduce selection bias and confounding, although statistical adjustments will be applied where possible. The lack of blinding for participants and acupuncturists may lead to performance bias, though objective endpoints will help mitigate this. The absence of a sham acupuncture control also means that placebo effects cannot be fully discounted. Finally, the use of specific acupoint protocols may limit the generalizability of findings to other acupuncture techniques or point selections.

In conclusion, this study addresses an important gap in the current evidence base by examining the real-world effectiveness of manual acupuncture for CNP in the elderly. By focusing on an aging population with high clinical need and employing a pragmatic comparative design, this research has the potential to inform integrative treatment strategies and support evidence-based decision-making in geriatric pain management.
